# Early Life Stress as a Predictor of Co-Occurring Alcohol Use Disorder and Post-Traumatic Stress Disorder

**DOI:** 10.35946/arcr.v39.2.05

**Published:** 2018

**Authors:** Richard S. Lee, Lynn M. Oswald, Gary S. Wand

**Affiliations:** Richard S. Lee, Ph.D., is an assistant professor in the Department of Psychiatry and Behavioral Sciences, Johns Hopkins University School of Medicine, Baltimore, Maryland. Lynn M. Oswald, Ph.D., R.N., is an associate professor in the Department of Family and Community Health, University of Maryland School of Nursing, Baltimore, Maryland. Gary S. Wand, M.D., is a professor in the Departments of Psychiatry and Behavioral Sciences and Medicine, Johns Hopkins University School of Medicine, Baltimore, Maryland

**Keywords:** addiction, alcohol use disorder, animal models, genotype, post-traumatic stress disorder, psychological stress

## Abstract

During the critical developmental periods of childhood when neural plasticity is high, exposure to early life stress (ELS) or trauma may lead to enduring changes in physiological stress systems and enhanced vulnerability for psychopathological conditions such as post-traumatic stress disorder (PTSD) and alcohol use disorder (AUD) in adulthood. Clinical and preclinical studies have sought to understand the possible mechanisms linking ELS, PTSD, and AUD. Preclinical studies have employed animal models of stress to recapitulate PTSD-like behavioral deficits and alcohol dependence, providing a basic framework for identifying common physiological mechanisms that may underlie these disorders. Clinical studies have documented ELS-related endocrine dysregulation and genetic variations associated with PTSD and AUD, as well as disruption in crucial neural circuitry throughout the corticomesolimbic region. Despite limitations and challenges, both types of studies have implicated three interrelated mechanisms: hypothalamic pituitary adrenal (HPA) axis and glucocorticoid signaling dysregulation, genetics, and epigenetics. ELS exposure leads to disruption of HPA axis function and glucocorticoid signaling, both of which affect homeostatic cortisol levels. However, individual response to ELS depends on genetic variations at specific genes that moderate HPA axis and brain function, thus influencing susceptibility or resilience to psychopathologies. Epigenetic-influenced pathways also are emerging as a powerful force in helping to create the PTSD and AUD phenotypes. Dysregulation of the HPA axis has an epigenetic effect on genes that regulate the HPA axis itself, as well as on brain-specific processes such as neurodevelopment and neurotransmitter regulation. These studies are only beginning to elucidate the underpinnings of ELS, PTSD, and AUD. Larger human cohorts, identification of additional genetic determinants, and better animal models capable of recapitulating the symptoms of PTSD and AUD are needed.

## Overview

Although various forms of stress experienced during adulthood can be antecedents for the onset of alcohol use disorder (AUD) and post-traumatic stress disorder (PTSD), stressful events suffered during childhood may produce mechanistically distinct changes in the developing nervous system that increase lifelong risks for the co-occurrence of both disorders.^1^ Early life stress (ELS) has been characterized as any form of severe trauma experienced before age 18 that could lead to pathological consequences in adulthood.^2^ The trauma may have resulted from maltreatment, such as sexual, physical, or emotional abuse; or stressful life events, such as loss of a parent, economic adversity, or family violence.

Unfortunately, childhood maltreatment is all too common. In 2014, child protective service agencies received an estimated 3.6 million referrals involving approximately 6.6 million children.^3^ Roughly, 702,000 of these referrals, 9.4 out of 1,000 children nationally, were considered victims of maltreatment (abuse or neglect). Percentages were similar for boys (48.9%) and girls (50.7%). However, for children younger than age 6, percentages for boys were consistently larger than they were for girls, whereas for older age groups, percentages for girls were larger than they were for boys. Although these numbers are appalling, they likely represent only the tip of the iceberg, as they do not include cases that go unreported or unverified and do not include other forms of ELS.

There has been growing awareness that the consequences of ELS extend beyond immediate effects, such as fear, injury, or isolation, to include lifelong ramifications on risks for an array of physical (e.g., cardiovascular disease, cancer, diabetes, fractures, and autoimmune disorders) and mental health (e.g., depression, anxiety, PTSD, and substance use disorder) problems, as well as on symptom severity and response to treatment. The idea that such effects could be a result of ELS-induced, long-term alterations in the central nervous system and other biological systems was initially met with some resistance in the scientific community.^4^ However, a robust body of evidence now supports the validity of such hypotheses. Findings from a growing number of studies, beginning with the landmark Adverse Childhood Experiences study, suggest that there is a “dose-response” relationship between ELS and adult pathology, such that greater trauma is associated with greater risks for negative sequelae.^5^ Moreover, studies of ELS report significant gender-specific prevalence, not only in the types and durations of trauma exposure, but also in rates of psychiatric outcomes such as depression, dissociation, and PTSD.^6^ Studies also report physiological consequences, such as reduced hippocampal volume.^7^ In general, findings of clinical studies suggest that ELS-induced sequelae are more severe in females than in males, and preclinical studies support this notion.^8^

ELS increases the risk for a variety of adulthood psychiatric and metabolic disorders, but it has a particularly powerful influence on the emergence of AUD and PTSD. Not only are individuals who lived through significant ELS at high risk for developing AUD, but they also have increased risk of a more severe form of the disorder characterized by early age of onset.^9^ The increased risk for AUD associated with early childhood maltreatment remains sustained into middle life,^10^ implicating long-term changes in key neural circuitry regulating the stress response and the reward systems. Studies have also shown that the risk for developing AUD in adulthood correlates with the number of adverse childhood experiences endured.^11^ This dose-dependent effect (severity and frequency) of stress can result from an acute and toxic exposure but is often the consequence of chronic maltreatment.^12^ Typically, these individuals have been exposed to multiple and varied types of abuse.^13^ Although all forms of significant trauma and abuse (physical, sexual, and emotional) during childhood can precede the development of AUD, sexual abuse appears to be one of the more potent risk factors.^14^

The fifth edition of the *Diagnostic and Statistical Manual of Mental Disorders* reclassified PTSD as a trauma-related disorder rather than an anxiety disorder. This new grouping recognizes that the array of symptoms associated with PTSD emerges only after exposure to a significant traumatic event. In addition to increasing the risk for AUD, the types of trauma falling under the definition of ELS can increase vulnerability for the development of PTSD.^15^ Therefore, it is not surprising that a number of studies have found high co-occurrence of AUD and PTSD.^16^,^17^ A review by Shorter and colleagues identified that alcohol is the most commonly misused drug among individuals with PTSD.^18^ Other researchers have noted that the severity and number of childhood abusive episodes are associated with the prevalence of AUD and the gravity of PTSD symptoms, once again indicating a dose effect of stress.^19^ A large epidemiological study showed that the risk of AUD was increased in women with a history of ELS, when compared with women who had no such history, but a history of trauma resulting in PTSD increased the risk for AUD almost twofold, indicating an additive effect on risk.^20^ It is assumed that PTSD precedes the development of AUD in most individuals with comorbid disorders.^15^ This hypothesis makes sense, given that many of the symptoms of PTSD (e.g., hypervigilance, insomnia, flashbacks, and lability of mood) are mitigated by the sedative effects of alcohol.

In this review, we examine some of the relevant preclinical models that address the effect of ELS on PTSD-like behavioral deficits and on alcohol consumption. We then integrate existing findings from preclinical and clinical literature to offer several potential mechanisms that may play a central role in the transition from ELS to later development of PTSD and AUD. These emerging findings provide evidence that genetic variation, epigenetic modulation of certain “stress” genes, and sustained alterations in hypothalamic pituitary adrenal (HPA) axis dynamics contribute to risks for PTSD and AUD in people who have a history of ELS.

## Preclinical Models

Preclinical animal models have been indispensable in terms of providing access to brain tissues and circuits, minimizing confounding factors, and enabling the examination of behavioral phenotypes associated with ELS, PTSD, and AUD. In particular, to identify molecular substrates that directly contribute to disease symptoms, researchers can examine the brain in close detail for candidate genes and for epigenetic and other mechanisms within specialized neural circuits. However, animal models may lack validity for modeling the human condition.

A vast number of studies have examined animal facsimiles of human stress or alcohol administration, but the types of stressors, trauma, and alcohol exposure differ (see Gilpin and Weiner for a review).^15^ The ideal model would be a paradigm of ELS that can manifest symptoms consistent with human PTSD, and the animals engage in increased alcohol consumption. However, creating models in which alcohol-naïve animals increase consumption following acute or chronic stress exposure is challenging. Researchers have been more successful using models in which animals resume alcohol consumption following a period of alcohol dependence, brief abstinence, and then stress exposure. Also, most researchers have used stress paradigms in adult rodents rather than in pups.

Currently, few promising paradigms exist. Because of the onus of documenting the relevant behavioral, biochemical, and neuroendocrine factors associated with ELS, PTSD, and AUD, no single study has successfully identified all facets of the interrelationships and causality among the three conditions. Instead, investigators have used animal models to examine different features of the three phenotypes. For example, in two studies of adult animals, exposure to predatory odors produced highly stress-reactive rats that increased their alcohol consumption.^21^,^22^ In another study, experiments using mice showed that a repetitive forced swim test coupled with chronic, intermittent, alcohol vapor exposure escalated alcohol consumption.^23^

Social isolation studies imposed on adolescent rats are very relevant to a link between ELS and AUD. Socially isolated adolescent rats have exhibited a wide range of behavioral changes, such as anxietylike behavior,^24^ sensory gating impairment,^25^ hyperactivity in a novel environment,^26^ and deficits in fear extinction,^27^ all of which are component behaviors associated with PTSD. These behavioral impairments can persist from adolescence into adulthood, as was demonstrated in a study in which rats that were socially isolated as adolescents increased their alcohol intake as adults, when compared with group-housed counterparts.^27^ In other studies, alcohol intake,^28^ alcohol preference,^29^ and PTSD-associated symptoms^30^,^31^ such as anxiety, sensory impairments, and fear extinction deficits were observed in socially isolated adolescent mice.

Only a few studies have focused on an earlier developmental period. One study induced stress in rats through maternal separation and then examined alcohol intake during adolescence.^32^ In this study, adolescent alcohol intake was exacerbated by additional stress exposure. However, it is unclear whether these maternally separated animals developed other PTSD-related behavioral deficits, such as those exhibited by rats in the social isolation studies.

A common theme that emerges from these animal stress models is that exposure to stress, especially during early development, leads to a number of anxiety- and PTSD-like behavioral deficits that persist for some time throughout development. Further, in some of the studies, the animals either escalated or resumed alcohol intake, serving as promising models for examining the physiological processes and other underlying mechanisms that link stress exposure to alcohol consumption.

## Potential Mechanisms

The disruption of substantially overlapping circuitries is central to preclinical and clinical research on the mechanisms through which ELS contributes to PTSD and AUD. In this section, we examine HPA axis and glucocorticoid signaling, genetic variations, and epigenetic mechanisms. These interrelated mechanisms may underlie the comorbid symptomatology that characterizes PTSD and AUD. Although it is possible that the relationships among ELS, PTSD, and AUD can be mediated by glucocorticoid-independent mechanisms, we consider the mechanisms in the context of glucocorticoid signaling.

### The HPA axis and glucocorticoid signaling

The HPA axis is the key neuroendocrine component of the stress response. Release of corticotropin-releasing hormone (CRH) and adrenocorticotropic hormone (ACTH) neuropeptides from the hypothalamus and the pituitary, respectively, culminates in the release of the stress hormone cortisol (or corticosterone in rodents) from the adrenal cortex. Cortisol is a glucocorticoid that, in addition to its primary role in the release of stored glucose during the fight-or-flight response, targets a number of cellular processes by binding to the glucocorticoid receptor encoded by the nuclear receptor subfamily 3 group C member 1 gene, *NR3C1*. Negative feedback mechanisms in brain regions such as the hippocampus and the prefrontal cortex (PFC), and positive feedback mechanisms in the amygdala, dampen or amplify the HPA axis, respectively. There has been substantial focus on the HPA axis and glucocorticoid signaling, because normal function is dysregulated in individuals exposed to ELS and in those with AUD and PTSD.^33^ Glucocorticoids have also attracted attention in the pathophysiology of ELS, PTSD, and AUD, because glucocorticoid signaling is involved in some forms of learning consolidation and memory formation, as well as in emotion regulation and reward reinforcement.

The consequences of glucocorticoid signaling follow an inverted U-shaped function in which extremely high and extremely low levels can be detrimental.^34^ Both extremes are observed in people who have experienced ELS and in those with PTSD and AUD. The high concentrations of glucocorticoids achieved during the early phase of ELS lead to profound and durable changes in HPA axis function and in hypothalamic and extrahypothalamic CRH expression. For example, in studies that used maternal deprivation models in which rats were separated from their mothers for up to 24 hours, or macaques were raised without their mothers after age 6 months, the animals showed increased concentrations of the stress peptide CRH that persisted into adulthood within the mesolimbic system (e.g., in the amygdala) and cerebrospinal fluid.^35^–^37^ These allostatic modifications were associated with marked increases in anxietylike behavior. Given that amygdala CRH neurons are known targets of glucocorticoid signaling, it is not surprising that altered *NR3C1* gene expression has been observed in this region.

Findings of several studies now indicate that ELS-related behavioral changes in rodents can be prevented or normalized with glucocorticoid receptor or CRH type 1 receptor antagonists.^38^–^40^ A glucocorticoid receptor antagonist has also been shown to decrease amygdala activation in rats undergoing a forced swim test, a result consistent with inhibition of central stress activation.^41^ In addition, elevated CRH in cerebrospinal fluid has been observed in people who have experienced ELS. For participants in one study, CRH levels were correlated with scores on the Childhood Trauma Questionnaire, particularly with emotional neglect.^42^

Dysregulation of cortisol levels is often associated with ELS. However, whether ELS exposure leads to high or low cortisol levels remains inconclusive. Low levels may occur more frequently in individuals who experienced ELS episodes more often or with more severity. However, enhanced sensitivity to glucocorticoid negative feedback and blunted cortisol responses to acute stress have been reported.^43^

Similar to what has been demonstrated in rodent models, human behavioral manifestations of ELS often mimic mood and anxiety states, including hyperresponsiveness of limbic regions, hyporesponsiveness of prefrontal regions that regulate limbic responses, and decreased engagement of striatal regions involved in reward processing. Both the amygdala and medial PFC (mPFC) are particularly affected by ELS. Most neuroimaging studies of people who have experienced ELS show an increased amygdala volume and hyperresponsivity, both of which have been associated with increased trait anxiety and diminished reward sensitivity.^44^ Other research has demonstrated that adults who experienced ELS have reduced mPFC volume^45^ and reduced mPFC activation during cognitive tasks.^46^

PTSD and AUD are also associated with persistent alterations in HPA axis dynamics. The HPA axis dysfunction observed in individuals with PTSD is characterized by a state of low basal glucocorticoid levels and increased sensitivity to glucocorticoids.^47^ This pattern mirrors findings observed in those who have experienced multiple episodes of ELS.^48^ These modifications in stress pathways may be mechanistically related to the symptoms of PTSD. However, in a recent clinical trial, the glucocorticoid receptor antagonist mifepristone was not demonstrated to be an effective treatment for Gulf War veterans.^49^ The treatment consisted of a 6-week phase both before and after a 1-month washout period. The researchers determined that the mifepristone treatment did not affect neurocognitive functioning or self-reported physical health, depression, PTSD symptoms, or fatigue. Therefore, it remains uncertain whether alterations in glucocorticoid signaling are fundamentally related to the PTSD phenotype.

HPA axis dynamics in AUD are modified as a function of alcohol consumption, withdrawal, and abstinence. In individuals who have AUD, glucocorticoid levels are high during episodes of drinking and acute withdrawal from alcohol.^33^ During prolonged periods of abstinence from alcohol, glucocorticoid levels may be low in the unstressed state and following stressful stimulation.^50^,^51^ In contrast, individuals with a history of ELS or PTSD exhibit low glucocorticoid levels and enhanced sensitivity to glucocorticoid negative feedback.^52^

The magnitude of alcohol activation of dopamine reward circuitry is considered an early mechanism for accelerating alcohol consumption. However, in more severe forms of AUD, the emergence of stress peptide expression may become the dominant mechanism for provoking alcohol cravings and alcohol-seeking behavior. In rodent models of AUD, there is an allostatic shift in CRH expression in the central amygdala. The advent of increased CRH expression is associated with anxietylike behavior, which has been called the “dark side” of AUD pathogenesis.^53^

A similar mechanism is at work in people with AUD, causing dysphoria and craving rather than dopamine-induced pleasure and reward. Alcohol’s modulation of the HPA axis coupled with its sedative properties are possibly causally related to and compensatory for both ELS-related trauma and PTSD. Although this theory may be premature, it is supported by the candidate gene studies discussed in the next section.

### Genetic variations

In addition to dysregulated HPA axis function and glucocorticoid signaling, genetics are a mechanism that could link ELS to PTSD and AUD. Specifically, DNA sequence variations are believed to contribute to an individual’s response to ELS and serve as risk or resilience factors for the development of PTSD or AUD symptoms. At the molecular level, these variations alter protein activity through changes in the encoded peptide sequence. The variations can also affect gene expression levels by altering gene activation mediated by transcription factor binding. In general, variations relevant to ELS, PTSD, or AUD are found in genes with encoded proteins that regulate glucocorticoid signaling, neurotransmitter regulation, or alcohol metabolism. It is believed that disease is precipitated by alterations in protein function or gene activation, which are moderated by these genetic variations. Glucocorticoid-related and epigenetic mechanisms associated with trauma exposure can also result in changes in gene function.

Genetic risk factors are innate and inherited. Transgenerational inheritance of epigenetic modifications related to ELS, PTSD, or AUD is an active area of research. The heritability for PTSD following exposure to trauma ranges from 24% to 72%, and the heritability percentage for women is larger than the percentage for men.^54^ A 2002 meta-analysis of 50 family, twin, and adoption studies indicated an upper limit of 30% to 36% for AUD heritability.^55^ A more recent meta-analysis that examined twin and adoption studies showed the heritability of AUD to be an estimated 50%, with a modest proportion (10%) attributed to shared environmental factors.^56^

Genetic research examining the molecular underpinnings of PTSD and AUD includes both hypothesis-driven, candidate gene association studies and unbiased, genome-wide approaches. Researchers have used both of these methods to identify variations at specific genomic loci associated with PTSD or AUD.

#### Candidate gene association studies

In candidate gene association studies, genes related to neurotransmitter regulation, alcohol metabolism, and the stress response (HPA axis) have been examined. Small candidate gene association studies of trauma survivors with and without PTSD have implicated the tandem repeat sequence of the dopamine transporter gene, *SLC6A3*,^57^ and a functional insertion/deletion within the serotonin transporter gene, *SLC6A4*.^58^ In addition, a single nucleotide polymorphism (SNP) within the putative estrogen receptor binding site in the stress response gene encoding the pituitary adenylate cyclase activating polypeptide (*ADCYAP1*) has been shown to be associated with PTSD diagnosis and symptoms in women.^59^ In other studies, although statistically significant associations with PTSD were lacking, SNPs associated with *NR3C1*^60^ and FK506 binding protein 5 (*FKBP5*)^61^ have been shown to interact with trauma exposure to predict the severity of PTSD symptoms.

Several notable AUD studies have examined catechol-O-methyltransferase (*COMT*),^62^ gamma-aminobutyric acid type A receptor alpha2 subunit (*GABRA2*),^63^ cholinergic receptor muscarinic 2 (*CHRM2*),^64^ and several genes involved in alcohol metabolism.^65^ Other studies have attempted to assess whether candidate SNPs can moderate the effect of stress or trauma exposure on AUD. Blomeyer and colleagues found that an interaction between an intronic SNP in the corticotropin releasing hormone receptor 1 (*CRHR1*) gene and stressful life events predicted heavy alcohol use.^66^ Another study of the interaction between *CRHR1* SNPs and adult traumatic stress exposure showed a significant effect on the likelihood of developing AUD.^67^ Similarly, in other research, women who experienced childhood sexual abuse and who carried the low-activity allele of the monoamine oxidase A (*MAOA*) gene had significantly higher rates of AUD, when compared to control subjects.^68^

Some researchers have employed gene knock-in or knockout strategies in mice to assess the functional consequences of genetic variations identified in humans. A mouse knock-in study of the Val68Met SNP in the human brain derived neurotrophic factor (*BDNF*) gene, which is regulated by glucocorticoids, showed that introduction of the Met68BDNF allele dramatically increased alcohol consumption.^69^ In a functional study of the *FKBP5* gene, researchers examined the effect of SNPs that are significantly associated with severity of alcohol withdrawal symptoms by knocking out the gene in mice.^70^ In an analysis of human subjects, researchers determined that one of the same SNPs influenced allele-specific epigenetic modifications following exposure to ELS.^71^ A study of healthy individuals showed that several of these SNPs were associated with differential cortisol responses to stress, strongly supporting their role in glucocorticoid signaling and HPA axis function.^72^ Together, these studies demonstrate that genetic variations that potentially affect gene function can moderate the effect of stress or trauma on AUD.

#### Genome-wide association studies

Over the past 10 years, genome-wide association studies with large cohort sizes have gained traction because they can provide statistical power and an unbiased approach to uncovering novel genomic loci associated with a disease. However, in a 2017 genome-wide association study (*N* = 20,070), the Psychiatric Genomics Consortium for PTSD found no transethnic SNPs of genome-wide significance, although the researchers did find genetic overlap with schizophrenia.^54^ In fact, several studies have shown that psychiatric disorders and PTSD share genetic risk. Another recent genome-wide association study uncovered several loci associated with alcohol consumption, including several genes associated with alcohol metabolism.^73^ In addition, a 2017 analysis that used a polygenic score approach reported that AUD shared genetic susceptibility with depression.^74^

Currently, there are no genome-wide association studies of genetic variants that interact with ELS to precipitate PTSD and AUD. However, both genetic and genome-wide studies of PTSD and AUD have identified loci associated with neurotransmitter regulation, alcohol metabolism, and the HPA axis. Further, studies that examined genomic loci across different disorders found evidence for overlap of genetic risk factors for PTSD, AUD, and other psychiatric disorders. This genetic overlap becomes especially relevant in understanding the epigenetic mechanisms associated with PTSD and AUD and helps us understand ELS-induced comorbidities in the larger context of psychiatric and substance use disorders.

### Epigenetic mechanisms

In general, epigenetics refers to DNA, DNA-associated histone protein, or noncoding RNA modifications that can coordinate sustained gene regulation without changing the underlying DNA sequence. The detrimental effect of ELS on the human brain cannot be fully captured by the permanent information encoded by DNA. Physiological consequences of ELS may be mediated by epigenetic mechanisms, since ELS can lead to prolonged changes in gene function without changing the DNA sequence. The early-life exposure event in conjunction with genetic susceptibility is believed to lead to long-lasting changes in gene function to precipitate symptoms of PTSD and AUD in adulthood.

A number of epigenetic studies have examined the molecular consequences of exposure to stress or glucocorticoids, with potential implications for PTSD and AUD. Glucocorticoid signaling, which can directly alter epigenetic marks via glucocorticoid receptors, is one of the central mechanisms that enables stress-related events to alter brain function. Studies have demonstrated that chronic glucocorticoid exposure or isolation stress can lead to long-lasting loss of DNA methylation at *Fkbp5*^75^ and tyrosine hydroxylase (*Th*) in vivo,^76^ respectively, as well as at hundreds of loci across the genome.^77^

Exposure to ELS or glucocorticoids has also been shown to lead to epigenetic alterations of genes such as *CRH*, *NR3C1*, and *FKBP5*. Epigenetic regulation of these glucocorticoid target genes is noteworthy and has long-term implications, given their prominent role in HPA axis function. For instance, it has been well-established that genetic and epigenetic variations in the *NR3C1* and *FKBP5* genes contribute to hypercortisolemia and glucocorticoid resistance, because changes in *NR3C1* and *FKBP5* gene expression directly affect extracellular glucocorticoid levels and intracellular glucocorticoid signaling.^78^

Another group of glucocorticoid targets consists of genes that control tissue-specific processes. Genes that are expressed in the brain and are involved in neurodevelopment and neurotransmission are relevant to ELS, PTSD, and AUD. ELS-induced, long-term disruption of HPA axis function and epigenetic regulation of genes such as *NR3C1* and *FKBP5*, in turn, can affect epigenetic regulation of the *BDNF*, *TH*, and *MAOA* genes. These glucocorticoid target genes are critical for neurodevelopment and neurotransmitter function and, along with the glucocorticoid signaling genes *NR3C1* and *FKBP5*, can serve as molecular substrates that link ELS exposure and behavioral disorders such as PTSD, AUD, and substance use disorder. A causal relationship between glucocorticoid exposure and risk for psychiatric disorders is strongly supported by findings from large epidemiological studies.^79^

In the overall framework proposed, ELS disrupts homeostatic glucocorticoid levels in the system via epigenetic changes at specific genes that regulate glucocorticoid signaling. This disruption of homeostasis, in turn, leads to alterations of genes that precipitate psychiatric symptoms. Many of the genes that are epigenetically modified by ELS also play prominent roles in AUD and PTSD.

Epigenetics research on candidate genes that mediate the effect of ELS on PTSD and AUD is scarce. In this section we discuss the research on several genes in the context of stress, PTSD, or AUD, including studies that used human cohorts and those that used animal models of stress and alcohol intake. We briefly discuss six genes, *CRH*, *NR3C1*, *FKBP5*, *BDNF*, *MAOA*, and *TH*, to exemplify how ELS can epigenetically alter gene function, which then potentially can affect behavioral symptoms, such as those observed in PTSD and AUD. For individuals who have experienced ELS and PTSD, alcohol use may induce gene expression and epigenetic changes to compensate for gene expression and epigenetic deficits.

#### *CRH* gene

*CRH* is a gene that has been implicated in ELS, PTSD, and AUD. It acts as one of the primary determinants of the brain’s stress response and alcohol dependence. In adult mice, social defeat stress has been associated with a decrease in methylation at the *Crh* promoter in the paraventricular nucleus.^80^ This finding is supported by studies that reported increased CRH levels in the cerebrospinal fluid and plasma of individuals with PTSD.^81^–^83^ In other studies, adult rodents and nonhuman primates that were deprived of their mothers during youth have shown increased CRH concentrations within and outside the hypothalamus and in the cerebrospinal fluid.^35^–^37^ These animals may exhibit hyperactive HPA axis and behavioral stress responses throughout life. As mentioned previously, elevated CRH in cerebrospinal fluid has also been observed in humans who have a history of ELS.^42^

CRH plays a critical role in AUD. Administration of CRH type 1 receptor antagonists in mice has been shown to attenuate alcohol-seeking behavior and withdrawal-induced drinking,^84^,^85^ although such observations have not been strongly supported in human studies. As with stress exposure, alcohol administration activates the HPA axis, inducing release of CRH, ACTH, and cortisol. CRH production in the amygdala increases with chronic alcohol administration, resulting in long-term upregulation of *CRHR1* gene expression in specific regions of the brain. One of the mechanisms that potentiates alcohol-seeking behavior following exposure to ELS may be transactivation of the *CRH* gene resulting from a loss of methylation at its promoter.

#### *NR3C1* gene

The *NR3C1* gene encodes the primary receptor for binding cortisol, and this receptor is believed to be responsible for the detrimental effects of HPA axis dysregulation. Recent evidence has implicated glucocorticoid signaling as a prominent factor in AUD and in many aspects of other substance use disorders.^86^,^87^ In research relevant to ELS, poor maternal nursing behavior in rats has been shown to alter adulthood HPA axis function, as indicated by an increase in DNA methylation at one of the *Nr3c1* promoters.^88^ In a study that examined human cord blood, researchers suggested that a similar mechanism developed in infants exposed in utero to maternal depression.^89^

In contrast, one study has documented different epigenetic patterns in individuals with PTSD, with those participants exhibiting a reduction in overall methylation and an increase in *NR3C1* expression, which enhances glucocorticoid trafficking.^90^ In another study that compared individuals with PTSD to healthy controls, those with PTSD had consistently lower baseline cortisol levels, and they had a greater ability to suppress cortisol levels following a dexamethasone suppression test.^47^ Although the molecular transition in glucocorticoid receptor sensitivity from ELS to PTSD is unclear, it is likely dependent on the type and duration of ELS. Further, the elevated cortisol levels achieved during alcohol intoxication may be compensating for hyperreactive glucocorticoid signaling and lower cortisol levels.

#### *FKBP5* gene

*FKBP5* is another gene that plays a crucial role in regulating systemic and intracellular glucocorticoid signaling. It encodes a chaperone protein that tethers the glucocorticoid receptor and prevents downstream glucocorticoid signaling, thereby attenuating glucocorticoid sensitivity. A study of primates implicated *FKBP5* as one of the main determinants of glucocorticoid resistance.^78^ A study in humans examined gene-environment interaction between a risk allele associated with enhanced gene expression and ELS exposure.^71^ The researchers reported that ELS-exposed, risk-allele carriers showed loss of intronic methylation near a glucocorticoid response element that affected glucocorticoid-induced activation of *FKBP5*. Another study reported that *FKBP5* alleles interacted with ELS to increase the risk for PTSD.^91^

ELS-induced modulation of *FKBP5* expression also has important implications for AUD. In preclinical studies, *Fkbp5* expression levels modulated alcohol intake and withdrawal severity, with *Fkbp5* knockout mice increasing alcohol intake and exhibiting sensitivity to alcohol withdrawal.^70^,^92^ In humans, a study has linked a SNP genotype of *FKBP5* and the presence of poor child-parent relationships to problematic drinking behavior.^93^ Collectively, ELS exposure leads to epigenetic changes at genes that alter HPA axis function, and those changes, along with genetic variations, may increase the risk for the development of PTSD. Although the molecular transition that takes place from ELS exposure to PTSD is still unclear, the effect of ELS exposure on glucocorticoid signaling is associated with increased alcohol intake and withdrawal severity.

#### *BDNF* gene

In addition to genes that regulate the HPA axis and glucocorticoid signaling, downstream glucocorticoid receptor target genes that regulate brain-specific processes also have a significant effect on ELS-induced behavior. As a member of the neurotrophin family of growth factors, the BDNF protein promotes neuronal survival, protection, and growth, as well as synaptic plasticity and neurotransmission.

A well-studied SNP, the Val66Met polymorphism, has been shown to interact with ELS to predict symptoms consistent with depression, anxiety, and cognitive decline.^94^ In rodent models, stress exposure in many forms and during several developmental periods leads to a decrease in *Bdnf* expression via epigenetic mechanisms. For example, maternal separation or early weaning has been shown to lead to decreased expression by promoting histone deacetylation at exon IV,^95^ social isolation has been associated with an increase in intronic glucocorticoid response element DNA methylation during adolescence,^96^ and social defeat has been linked to histone deacetylation during adulthood.^97^

Similar findings have been observed in individuals with PTSD. In one study, a meta-analysis implicated the Val66Met polymorphism in trauma-exposed individuals with PTSD.^98^ Researchers have reported that in veterans with PTSD, when compared to veterans without PTSD, peripheral BDNF protein levels were lower, and DNA methylation in the gene promoter was higher.^99^ For the *BDNF* gene, alcohol appears to compensate for ELS- or PTSD-induced deficiencies, as demonstrated by a study in which acute alcohol administration led to histone acetylation-associated increases in the central and medial amygdala of alcohol-preferring rats.^100^

#### *MAOA* and *TH* genes

The *MAOA* gene encodes an enzyme that oxidizes and breaks down monoamine neurotransmitters such as dopamine, serotonin, and adrenaline. Of these monoamine neurotransmitters, dopamine has garnered the most interest regarding alcohol and substance misuse because of its involvement in stress and reward pathways. The *TH* gene encodes the rate-limiting enzyme involved in the synthesis of dopamine, tyrosine hydroxylase. Both the *MAOA* and *TH* genes are regulated by glucocorticoids.^96^,^101^,^102^ Through glucocorticoid-mediated, epigenetic dysregulation of dopamine function, these genes provide the means for ELS exposure to increase risk for the development of PTSD and AUD.

In a study using an animal model, exposure to peripubertal stress increased *Maoa* gene expression in the prefrontal cortex of rats, supported by an increase in histone H3 acetylation at the gene promoter.^103^ In another study, socially defeated mice showed a similar increase in the raphe nuclei.^104^ No studies have examined MAOA protein levels in relation to PTSD, but in one study of ELS-exposed rodents, alcohol exposure decreased MAOA activity and led to increased dopamine levels.^105^ In a study analyzing macaques, alcohol intake reduced expression levels of the *MAOA* gene in a dose-dependent manner.^106^

*TH* is another glucocorticoid target gene, and its expression levels are diminished in animals exposed to ELS.^96^ Although this gene has not been examined in the context of PTSD, *TH* expression levels have been increased by exposure to alcohol, providing yet another example of how alcohol use may be compensatory behavior to normalize gene function.^107^ A small study of pharmacological dopamine stimulation in humans showed enhanced reward-induced performance accuracy in participants who had poor parental care, further supporting the animal findings.^108^

## Future Research Needs

A brief review of the above candidate genes reflects the relative scarcity of data on the effects of ELS on comorbid PTSD and AUD, which necessitates additional investigations. First, an ELS model capable of recapitulating the component symptoms of both PTSD and AUD is needed. Animal model studies underscore the difficulty of modeling stress and alcohol exposure. Factors such as intensity, duration, and types of stress superimposed on different brain regions, circuits, and neurotransmitters have all contributed to different outcomes and further confounded conclusions. Development of robust animal models that can produce predicted phenotypical outcomes under standardized conditions is needed. Once established, these models can be implemented to examine the molecular underpinnings of PTSD and AUD. Use of genome-wide approaches can provide a bigger picture of relevant neuroadaptations, such as ELS-induced changes in specific pathways and gene sets. Specifically, genome-wide “omics” approaches, consisting of transcriptomics (RNA sequencing), epigenomics (methylation sequencing), and proteomics (mass spectrometry), can facilitate discovery and characterization of targets.

Similarly, human studies are lacking, except for a few clinical and candidate gene association studies. First and foremost, there is an urgent need for recruiting individuals who have comorbid AUD and PTSD rather than those who have AUD or PTSD alone, as underlying molecular mechanisms governing the comorbid condition may be unique and distinct. In addition, these cohorts need to be large enough to identify genetic variants that interact with ELS and are associated with PTSD and AUD. Once susceptibility genes and their variants have been identified, preclinical studies manipulating these genes can establish how the genes interact with ELS to precipitate PTSD and AUD symptoms. In addition, assays can be developed to identify individuals who may be predisposed genetically or epigenetically to PTSD and AUD.

Also, functional studies are needed to verify whether AUD is compensatory behavior to offset the molecular consequences of stress. Preclinical and clinical studies are needed to examine at the molecular level whether alcohol consumption can reverse many of the deficits caused by ELS exposure. Identification of such substrates of AUD can lead to development of medications that do not have the detrimental and addictive properties of alcohol.

Key questions that need to be addressed include:

- What mechanisms underlie the increased risks of developing AUD and PTSD following exposure to ELS?
- How do the allostatic changes that result from ELS remain durable over the lifetime of the individual?
- Why are only a subset of individuals at risk for AUD or PTSD following ELS?
- Are the allostatic changes that result from ELS both necessary and sufficient to produce the symptom complex associated with AUD and PTSD?
- Can these altered systems be targeted for therapeutic intervention?

## Conclusion

In this review, we sought to understand the mechanisms that underlie the link between ELS exposure and comorbid PTSD and AUD. Physiologically, the observed relationships are the result of ELS-induced, long-lasting, maladaptive changes in the stress and reward systems in the brain. Changes to these overlapping neural circuits have significant implications for PTSD and AUD. At the molecular level, a brief overview of several candidate genes suggests that ELS-induced epigenetic and transcriptional changes function as risk factors for AUD by promoting alcohol consumption.

Studies of genes such as *CRH* and *FKBP5* demonstrate that ELS-induced alterations in gene expression mimic the expression levels observed during alcohol intoxication, which may potentiate alcohol-seeking behaviors. Alternatively, studies of genes such as *NR3C1*, *BDNF*, *MAOA*, and *TH* suggest that alcohol consumption has an effect on gene expression and epigenetic regulation that may counteract the expression and epigenetic deficits caused by ELS. Therefore, alcohol consumption may be a coping behavior in an attempt to compensate for the molecular consequences of ELS.

The study of comorbid PTSD and AUD arising from ELS exposure is fertile ground for further investigation, as relatively few studies have been conducted. Additional animal model development; human studies; transcriptomic, epigenomic, and proteomic approaches; and specific therapeutic approaches are needed to understand and treat these debilitating psychiatric disorders.

## References

[1] Müller M, Vandeleur C, Rodgers S (2015). Childhood adversities as specific contributors to the co-occurrence of posttraumatic stress and alcohol use disorders. Psychiatry Res.

[2] Enoch MA (2011). The role of early life stress as a predictor for alcohol and drug dependence. Psychopharmacology (Berl).

[3] U.S. Department of Health and Human Services, Administration for Children and Families, Administration on Children, Youth and Families, Children’s Bureau (2016). Child Maltreatment 2014.

[4] Nemeroff CB (2016). Paradise lost:The neurobiological and clinical consequences of child abuse and neglect. Neuron.

[5] Felitti VJ, Anda RF, Nordenberg D (1998). Relationship of childhood abuse and household dysfunction to many of the leading causes of death in adults. The Adverse Childhood Experiences (ACE) Study. Am J Prev Med.

[6] Wamser-Nanney R, Cherry KE (2018). Children’s trauma-related symptoms following complex trauma exposure: Evidence of gender differences. Child Abuse Negl.

[7] Teicher MH, Anderson CM, Ohashi K (2018). Differential effects of childhood neglect and abuse during sensitive exposure periods on male and female hippocampus. Neuroimage.

[8] Brydges NM, Holmes MC, Harris AP (2015). Early life stress produces compulsive-like, but not impulsive, behavior in females. Behav Neurosci.

[9] Kaufman J, Yang BZ, Douglas-Palumberi H (2007). Genetic and environmental predictors of early alcohol use. Biol Psychiatry.

[10] Widom CS, White HR, Czaja SJ (2007). Long-term effects of child abuse and neglect on alcohol use and excessive drinking in middle adulthood. J Stud Alcohol Drugs.

[11] Anda RF, Felitti VJ, Bremner JD (2006). The enduring effects of abuse and related adverse experiences in childhood. A convergence of evidence from neurobiology and epidemiology. Eur Arch Psychiatry Clin Neurosci.

[12] Keyes KM, Hatzenbuehler ML, Hasin DS (2011). Stressful life experiences, alcohol consumption, and alcohol use disorders: The epidemiologic evidence for four main types of stressors. Psychopharmacology (Berl).

[13] Huang MC, Schwandt ML, Ramchandani VA (2012). Impact of multiple types of childhood trauma exposure on risk of psychiatric comorbidity among alcoholic inpatients. Alcohol Clin Exp Res.

[14] Kendler KS, Bulik CM, Silberg J (2000). Childhood sexual abuse and adult psychiatric and substance use disorders in women: An epidemiological and cotwin control analysis. Arch Gen Psychiatry.

[15] Gilpin NW, Weiner JL (2017). Neurobiology of comorbid post-traumatic stress disorder and alcohol-use disorder. Genes Brain Behav.

[16] Blanco C, Xu Y, Brady K (2013). Comorbidity of posttraumatic stress disorder with alcohol dependence among U.S. adults: Results from National Epidemiological Survey on Alcohol and Related Conditions. Drug Alcohol Depend.

[17] Debell F, Fear NT, Head M (2014). A systematic review of the comorbidity between PTSD and alcohol misuse. Soc Psychiatry Psychiatr Epidemiol.

[18] Shorter D, Hsieh J, Kosten TR (2015). Pharmacologic management of comorbid post-traumatic stress disorder and addictions. Am J Addict.

[19] Khoury L, Tang YL, Bradley B (2010). Substance use, childhood traumatic experience, and posttraumatic stress disorder in an urban civilian population. Depress Anxiety.

[20] Sartor CE, McCutcheon VV, Pommer NE (2010). Posttraumatic stress disorder and alcohol dependence in young women. J Stud Alcohol Drugs.

[21] Edwards S, Baynes BB, Carmichael CY (2013). Traumatic stress reactivity promotes excessive alcohol drinking and alters the balance of prefrontal cortex-amygdala activity. Transl Psychiatry.

[22] Manjoch H, Vainer E, Matar M (2016). Predator-scent stress, ethanol consumption and the opioid system in an animal model of PTSD. Behav Brain Res.

[23] Anderson RI, Lopez MF, Becker HC (2016). Forced swim stress increases ethanol consumption in C57BL/6J mice with a history of chronic intermittent ethanol exposure. Psychopharmacology (Berl).

[24] Yorgason JT, España RA, Konstantopoulos JK (2013). Enduring increases in anxiety-like behavior and rapid nucleus accumbens dopamine signaling in socially isolated rats. Eur J Neurosci.

[25] McCool BA, Chappell AM (2009). Early social isolation in male Long-Evans rats alters both appetitive and consummatory behaviors expressed during operant ethanol self-administration. Alcohol Clin Exp Res.

[26] Chappell AM, Carter E, McCool BA (2013). Adolescent rearing conditions influence the relationship between initial anxiety-like behavior and ethanol drinking in male Long Evans rats. Alcohol Clin Exp Res.

[27] Skelly MJ, Chappell AE, Carter E (2015). Adolescent social isolation increases anxiety-like behavior and ethanol intake and impairs fear extinction in adulthood: Possible role of disrupted noradrenergic signaling. Neuropharmacology.

[28] Lopez MF, Doremus-Fitzwater TL, Becker HC (2011). Chronic social isolation and chronic variable stress during early development induce later elevated ethanol intake in adult C57BL/6J mice. Alcohol.

[29] Holgate JY, Garcia H, Chatterjee S (2017). Social and environmental enrichment has different effects on ethanol and sucrose consumption in mice. Brain Behav.

[30] Varty GB, Powell SB, Lehmann-Masten V (2006). Isolation rearing of mice induces deficits in prepulse inhibition of the startle response. Behav Brain Res.

[31] Liu JH, You QL, Wei MD (2015). Social isolation during adolescence strengthens retention of fear memories and facilitates induction of late-phase long-term potentiation. Mol Neurobiol.

[32] Peñasco S, Mela V, Lopez-Moreno JA (2015). Early maternal deprivation enhances voluntary alcohol intake induced by exposure to stressful events later in life. Neural Plast.

[33] Stephens MA, Wand G (2012). Stress and the HPA axis: Role of glucocorticoids in alcohol dependence. Alcohol Res.

[34] Herman JP (2013). Neural control of chronic stress adaptation. Front Behav Neurosci.

[35] Ladd CO, Owens MJ, Nemeroff CB (1996). Persistent changes in corticotropin-releasing factor neuronal systems induced by maternal deprivation. Endocrinology.

[36] Plotsky PM, Thrivikraman KV, Nemeroff CB (2005). Long-term consequences of neonatal rearing on central corticotropin-releasing factor systems in adult male rat offspring. Neuropsychopharmacology.

[37] Coplan JD, Andrews MW, Rosenblum LA (1996). Persistent elevations of cerebrospinal fluid concentrations of corticotropin-releasing factor in adult nonhuman primates exposed to early-life stressors: Implications for the pathophysiology of mood and anxiety disorders. Proc Natl Acad Sci U S A.

[38] Arp JM, Ter Horst JP, Loi M (2016). Blocking glucocorticoid receptors at adolescent age prevents enhanced freezing between repeated cue-exposures after conditioned fear in adult mice raised under chronic early life stress. Neurobiol Learn Mem.

[39] Myers B, Greenwood-Van Meerveld B (2012). Differential involvement of amygdala corticosteroid receptors in visceral hyperalgesia following acute or repeated stress. Am J Physiol Gastrointest Liver Physiol.

[40] Prusator DK, Greenwood-Van Meerveld B (2017). Amygdala-mediated mechanisms regulate visceral hypersensitivity in adult females following early life stress: Importance of the glucocorticoid receptor and corticotropin-releasing factor. Pain.

[41] Wulsin AC, Herman JP, Solomon MB (2010). Mifepristone decreases depression-like behavior and modulates neuroendocrine and central hypothalamic-pituitary-adrenocortical axis responsiveness to stress. Psychoneuroendocrinology.

[42] Lee R, Geracioti TD, Kasckow JW (2005). Childhood trauma and personality disorder: Positive correlation with adult CSF corticotropin-releasing factor concentrations. Am J Psychiatry.

[43] Carpenter LL, Carvalho JP, Tyrka AR (2007). Decreased adrenocorticotropic hormone and cortisol responses to stress in healthy adults reporting significant childhood maltreatment. Biol Psychiatry.

[44] Thomason ME, Marusak HA (2017). Toward understanding the impact of trauma on the early developing human brain. Neuroscience.

[45] van Harmelen AL, van Tol MJ, van der Wee NJ (2010). Reduced medial prefrontal cortex volume in adults reporting childhood emotional maltreatment. Biol Psychiatry.

[46] van Harmelen AL, van Tol MJ, Dalgleish T (2014). Hypoactive medial prefrontal cortex functioning in adults reporting childhood emotional maltreatment. Soc Cogn Affect Neurosci.

[47] Yehuda R (2001). Biology of posttraumatic stress disorder. J Clin Psychiatry.

[48] Yehuda R (2010). Putative biological mechanisms for the association between early life adversity and the subsequent development of PTSD. Psychopharmacology (Berl).

[49] Golier JA, Caramanica K, Michaelides AC (2016). A randomized, double-blind, placebo-controlled, crossover trial of mifepristone in Gulf War veterans with chronic multisymptom illness. Psychoneuroendocrinology.

[50] Adinoff B, Iranmanesh A, Veldhuis J (1998). Disturbances of the stress response: The role of the HPA axis during alcohol withdrawal and abstinence. Alcohol Health Res World.

[51] Esel E, Sofuoglu S, Aslan SS (2001). Plasma levels of beta-endorphin, adrenocorticotropic hormone and cortisol during early and late alcohol withdrawal. Alcohol Alcohol.

[52] Daskalakis NP, Lehrner A, Yehuda R (2013). Endocrine aspects of post-traumatic stress disorder and implications for diagnosis and treatment. Endocrinol Metab Clin North Am.

[53] Koob GF (2015). The dark side of emotion: The addiction perspective. Eur J Pharmacol.

[54] Duncan LE, Ratanatharathorn A, Aiello AE (2018). Largest GWAS of PTSD (*N* = 20,070) yields genetic overlap with schizophrenia and sex differences in heritability. Mol Psychiatry.

[55] Walters GD (2002). The heritability of alcohol abuse and dependence: A meta-analysis of behavior genetic research. Am J Drug Alcohol Abuse.

[56] Verhulst B, Neale MC, Kendler KS (2015). The heritability of alcohol use disorders: A meta-analysis of twin and adoption studies. Psychol Med.

[57] Segman RH, Shalev AY (2003). Genetics of posttraumatic stress disorder. CNS Spectr.

[58] Lee HJ, Lee MS, Kang RH (2005). Influence of the serotonin transporter promoter gene polymorphism on susceptibility to posttraumatic stress disorder. Depress Anxiety.

[59] Ressler KJ, Mercer KB, Bradley B (2011). Post-traumatic stress disorder is associated with *PACAP* and the PAC1 receptor. Nature.

[60] Rohleder N, Joksimovic L, Wolf JM (2004). Hypocortisolism and increased glucocorticoid sensitivity of pro-inflammatory cytokine production in Bosnian war refugees with posttraumatic stress disorder. Biol Psychiatry.

[61] Binder EB, Bradley RG, Liu W (2008). Association of *FKBP5* polymorphisms and childhood abuse with risk of posttraumatic stress disorder symptoms in adults. JAMA.

[62] Tiihonen J, Hallikainen T, Lachman H (1999). Association between the functional variant of the catechol-O-methyltransferase (*COMT*) gene and type 1 alcoholism. Mol Psychiatry.

[63] Edenberg HJ, Dick DM, Xuei X (2004). Variations in *GABRA2*, encoding the alpha 2 subunit of the GABA_A_ receptor, are associated with alcohol dependence and with brain oscillations. Am J Hum Genet.

[64] Wang JC, Hinrichs AL, Stock H (2004). Evidence of common and specific genetic effects: Association of the muscarinic acetylcholine receptor M2 (*CHRM2*) gene with alcohol dependence and major depressive syndrome. Hum Mol Genet.

[65] Wall TL, Carr LG, Ehlers CL (2003). Protective association of genetic variation in alcohol dehydrogenase with alcohol dependence in Native American Mission Indians. Am J Psychiatry.

[66] Blomeyer D, Treutlein J, Esser G (2008). Interaction between *CRHR1* gene and stressful life events predicts adolescent heavy alcohol use. Biol Psychiatry.

[67] Ray LA, Sehl M, Bujarski S (2013). The *CRHR1* gene, trauma exposure, and alcoholism risk: A test of G × E effects. Genes Brain Behav.

[68] Ducci F, Enoch MA, Hodgkinson C (2008). Interaction between a functional *MAOA* locus and childhood sexual abuse predicts alcoholism and antisocial personality disorder in adult women. Mol Psychiatry.

[69] Warnault V, Darcq E, Morisot N (2016). The *Bdnf* valine 68 to methionine polymorphism increases compulsive alcohol drinking in mice that is reversed by tropomyosin receptor kinase B activation. Biol Psychiatry.

[70] Huang MC, Schwandt ML, Chester JA (2014). *FKBP5* moderates alcohol withdrawal severity: Human genetic association and functional validation in knockout mice. Neuropsychopharmacology.

[71] Klengel T, Mehta D, Anacker C (2013). Allele-specific *FKBP5* DNA demethylation mediates gene-childhood trauma interactions. Nat Neurosci.

[72] Mahon PB, Zandi PP, Potash JB (2013). Genetic association of *FKBP5* and *CRHR1* with cortisol response to acute psychosocial stress in healthy adults. Psychopharmacology (Berl).

[73] Clarke TK, Adams MJ, Davies G (2017). Genome-wide association study of alcohol consumption and genetic overlap with other health-related traits in U.K. Biobank (*N* = 112,117). Mol Psychiatry.

[74] Andersen AM, Pietrzak RH, Kranzler HR (2017). Polygenic scores for major depressive disorder and risk of alcohol dependence. JAMA Psychiatry.

[75] Lee RS, Tamashiro KL, Yang X (2010). Chronic corticosterone exposure increases expression and decreases deoxyribonucleic acid methylation of *Fkbp5* in mice. Endocrinology.

[76] Niwa M, Lee RS, Tanaka T (2016). A critical period of vulnerability to adolescent stress: Epigenetic mediators in mesocortical dopaminergic neurons. Hum Mol Genet.

[77] Seifuddin F, Wand G, Cox O (2017). Genome-wide methyl-seq analysis of blood-brain targets of glucocorticoid exposure. Epigenetics.

[78] Westberry JM, Sadosky PW, Hubler TR (2006). Glucocorticoid resistance in squirrel monkeys results from a combination of a transcriptionally incompetent glucocorticoid receptor and overexpression of the glucocorticoid receptor co-chaperone FKBP51. J Steroid Biochem Mol Biol.

[79] Fardet L, Petersen I, Nazareth I (2012). Suicidal behavior and severe neuropsychiatric disorders following glucocorticoid therapy in primary care. Am J Psychiatry.

[80] Elliott E, Ezra-Nevo G, Regev L (2010). Resilience to social stress coincides with functional DNA methylation of the *Crf* gene in adult mice. Nat Neurosci.

[81] Bremner JD, Licinio J, Darnell A (1997). Elevated CSF corticotropin-releasing factor concentrations in posttraumatic stress disorder. Am J Psychiatry.

[82] de Kloet CS, Vermetten E, Geuze E (2008). Elevated plasma corticotrophin-releasing hormone levels in veterans with posttraumatic stress disorder. Prog Brain Res.

[83] Baker DG, West SA, Nicholson WE (1999). Serial CSF corticotropin-releasing hormone levels and adrenocortical activity in combat veterans with posttraumatic stress disorder. Am J Psychiatry.

[84] Funk CK, Zorrilla EP, Lee MJ (2007). Corticotropin-releasing factor 1 antagonists selectively reduce ethanol self-administration in ethanol-dependent rats. Biol Psychiatry.

[85] Marinelli PW, Funk D, Juzytsch W (2007). The CRF1 receptor antagonist antalarmin attenuates yohimbine-induced increases in operant alcohol self-administration and reinstatement of alcohol seeking in rats. Psychopharmacology (Berl).

[86] Navarro-Zaragoza J, Hidalgo JM, Laorden ML (2012). Glucocorticoid receptors participate in the opiate withdrawal-induced stimulation of rats NTS noradrenergic activity and in the somatic signs of morphine withdrawal. Br J Pharmacol.

[87] Vendruscolo LF, Estey D, Goodell V (2015). Glucocorticoid receptor antagonism decreases alcohol seeking in alcohol-dependent individuals. J Clin Invest.

[88] Weaver IC, Cervoni N, Champagne FA (2004). Epigenetic programming by maternal behavior. Nat Neurosci.

[89] Oberlander TF, Weinberg J, Papsdorf M (2008). Prenatal exposure to maternal depression, neonatal methylation of human glucocorticoid receptor gene (*NR3C1*) and infant cortisol stress responses. Epigenetics.

[90] Labonté B, Azoulay N, Yerko V (2014). Epigenetic modulation of glucocorticoid receptors in posttraumatic stress disorder. Transl Psychiatry.

[91] Roy A, Gorodetsky E, Yuan Q (2010). Interaction of *FKBP5*, a stress-related gene, with childhood trauma increases the risk for attempting suicide. Neuropsychopharmacology.

[92] Qiu B, Luczak SE, Wall TL (2016). The *FKBP5* gene affects alcohol drinking in knockout mice and is implicated in alcohol drinking in humans. Int J Mol Sci.

[93] Nylander I, Todkar A, Granholm L (2017). Evidence for a link between *Fkbp5/FKBP5*, early life social relations and alcohol drinking in young adult rats and humans. Mol Neurobiol.

[94] Gatt JM, Nemeroff CB, Dobson-Stone C (2009). Interactions between *BDNF* Val66Met polymorphism and early life stress predict brain and arousal pathways to syndromal depression and anxiety. Mol Psychiatry.

[95] Seo MK, Ly NN, Lee CH (2016). Early life stress increases stress vulnerability through *Bdnf* gene epigenetic changes in the rat hippocampus. Neuropharmacology.

[96] Niwa M, Jaaro-Peled H, Tankou S (2013). Adolescent stress-induced epigenetic control of dopaminergic neurons via glucocorticoids. Science.

[97] Tsankova NM, Berton O, Renthal W (2006). Sustained hippocampal chromatin regulation in a mouse model of depression and antidepressant action. Nat Neurosci.

[98] Bruenig D, Lurie J, Morris CP (2016). A case-control study and meta-analysis reveal *BDNF* Val66Met is a possible risk factor for PTSD. Neural Plast.

[99] Kim TY, Kim SJ, Chung HG (2017). Epigenetic alterations of the *BDNF* gene in combat-related post-traumatic stress disorder. Acta Psychiatr Scand.

[100] Moonat S, Sakharkar AJ, Zhang H (2011). The role of amygdaloid brain-derived neurotrophic factor, activity-regulated cytoskeleton-associated protein and dendritic spines in anxiety and alcoholism. Addict Biol.

[101] Manoli I, Le H, Alesci S (2005). Monoamine oxidase-A is a major target gene for glucocorticoids in human skeletal muscle cells. FASEB J.

[102] Hagerty T, Morgan WW, Elango N (2001). Identification of a glucocorticoid-responsive element in the promoter region of the mouse tyrosine hydroxylase gene. J Neurochem.

[103] Marquez C, Poirier GL, Cordero MI (2013). Peripuberty stress leads to abnormal aggression, altered amygdala and orbitofrontal reactivity and increased prefrontal *Maoa* gene expression. Transl Psychiatry.

[104] Filipenko ML, Beilina AG, Alekseyenko OV (2002). Repeated experience of social defeats increases serotonin transporter and monoamine oxidase A mRNA levels in raphe nuclei of male mice. Neurosci Lett.

[105] Bendre M, Comasco E, Nylander I (2015). Effect of voluntary alcohol consumption on *Maoa* expression in the mesocorticolimbic brain of adult male rats previously exposed to prolonged maternal separation. Transl Psychiatry.

[106] Cervera-Juanes R, Wilhem LJ, Park B (2016). *MAOA* expression predicts vulnerability for alcohol use. Mol Psychiatry.

[107] Kawahata I, Evelyn GR, Huinan X (2017). Tyrosine hydroxylase gene expression is facilitated by alcohol followed by the degradation of the protein by ubiquitin proteasome system. Neuro Endocrinol Lett.

[108] Engert V, Joober R, Meaney MJ (2009). Behavioral response to methylphenidate challenge: Influence of early life parental care. Dev Psychobiol.

